# Vascular Endothelial Growth Factor, a Key Modulator of the Anti-Tumor Immune Response

**DOI:** 10.3390/ijms22094871

**Published:** 2021-05-04

**Authors:** Mannon Geindreau, François Ghiringhelli, Mélanie Bruchard

**Affiliations:** 1 Faculté des Sciences de Santé, Université Bourgogne Franche-Comté, 21000 Dijon, France; mannon.geindreau@u-bourgogne.fr; 2 Team “CAdIR”, CRI INSERM UMR1231 “Lipids, Nutrition and Cancer”, 21000 Dijon, France; 3 LipSTIC LabEx, 21000 Dijon, France; FGhirinchelli@cgfl.fr; 4 Centre Georges François Leclerc, 21000 Dijon, France

**Keywords:** vascular endothelial growth factor, angiogenesis, cancer, immune response

## Abstract

During tumor growth, angiogenesis is required to ensure oxygen and nutrient transport to the tumor. Vascular endothelial growth factor (VEGF) is the major inducer of angiogenesis and appears to be a key modulator of the anti-tumor immune response. Indeed, VEGF modulates innate and adaptive immune responses through direct interactions and indirectly by modulating protein expressions on endothelial cells or vascular permeability. The inhibition of the VEGF signaling pathway is clinically approved for the treatment of several cancers. Therapies targeting VEGF can modulate the tumor vasculature and the immune response. In this review, we discuss the roles of VEGF in the anti-tumor immune response. In addition, we summarize therapeutic strategies based on its inhibition, and their clinical approval.

## 1. Introduction

Since its discovery in the 1980s, vascular endothelial growth factor (VEGF) has attracted growing interest in the field of oncology. This molecule is involved in the formation of new blood vessels and was first seen upregulated in an aggressive form of glioblastoma [1]. Afterward, an antibody directed against VEGF (_A.4.6.1_) in mice was able to slow tumor growth in a wide range of tumors in vivo [2]. In 2004, Bevacizumab, an anti-VEGF-A, was the first human anti-angiogenic antibody approved for the treatment of colorectal cancer (CRC), and then approved in various diseases such as breast cancer (BC), non-small cell lung cancer (NSCLC), ovarian cancer (OC), and renal cell carcinoma (RCC) [3]. This review was achieved using PubMed and the following key words: VEGF, VEGF signaling pathway, angiogenesis, cancer, immune response, innate immune response, adaptive immune response, macrophages, natural killer, neutrophils, mast cells, myeloid-derived suppressor cells, dendritic cells, T-cells, therapy, therapy resistance.

## 2. Overview of VEGF Signaling Pathways and Its Major Contributors

During vasculogenesis and angiogenesis, VEGF plays a pivotal role; these two fundamental processes are involved in blood vessel formation. Vasculogenesis is the formation of a primitive vascular network during embryogenesis, whereas angiogenesis is the formation of new blood vessels from pre-existing vessels, appearing in physiological and some pathological processes such as cancer [4]. 

Angiogenesis is composed of different steps. When the process of angiogenesis is initiated, pre-existing blood vessels dilate, and pericytes detach from them. Basement membrane and extracellular matrix are degraded to allow the migration of endothelial cells in areas requiring new vessels. The basement membrane surrounds the vessel for cell and tissue support. Endothelial cells migrate and proliferate following an angiogenic stimuli such as VEGF. Lastly, these endothelial cells cluster together, forming a new basement membrane allowing the pericytes to cover the newly formed blood vessels [5].

The VEGF signaling pathways are indispensable during embryogenesis. Deficiencies in VEGF-A and VEGFR-2 elicit an abnormal vascular development resulting in an early embryogenic lethality in mice [6,7,8]. After birth, VEGF is involved in physiological events, such as pregnancy, growth, and menstrual cycles [9]. This protein also plays a prominent role in various pathological processes such as wound healing, retinopathy associated with blinding eye diseases, inflammatory diseases, and cancer [9]. 

When tissues are injured, angiogenesis, more specifically VEGF-A, is upregulated to form new capillaries in order to ensure nutriments, immune cells, and oxygen supply to the damaged area. After healing, this pro-angiogenic phase is followed by an anti-angiogenic process to return to a normal vessel density [10]. Targeting VEGF has dramatically improved the management of many diseases like blinding eye diseases such as age-related macular degeneration and diabetic and hypertensive retinopathy and shows promising data in cancer treatment [11]. In this review, we will focus on the roles of VEGFs in tumor development. 

The mammalian VEGF family is composed of five different glycoproteins: VEGF-A; VEGF-B; VEGF-C; VEGF-D; and Placental growth factor (PlGF). These proteins bind to three different vascular endothelial growth factor receptors (VEGFR): VEGFR1-3. These receptors are tyrosine kinase receptors [12]. 

After the binding of VEGF, VEGFR dimerizes with itself or with a co-receptor and auto-phosphorylates, leading to the activation of various intracellular signaling pathways [13]. These tyrosine kinase receptors are composed of three domains: an extracellular domain, a transmembrane region, and an intracellular tyrosine kinase domain [12]. There are two different co-receptors modulating the VEGFR signaling: neuropilin (NRP) and heparan sulfate proteoglycans (HSPG).

There are two NRP subtypes, NRP-1, and NRP-2. NRPs bind different VEGF isoforms. The proteins NRP-1 and NRP-2 are mainly expressed and associated with a poor clinical outcome in various tumors [14,15]. In vasculogenesis, NRP-1 plays a pivotal role as its deficiency results in an early embryogenic lethality in mice due to abnormal heart and vascular development as well as deficient neural guidance [16]. The binding of NRP-1 to VEGFR-2 increases the VEGFR-2/VEGF-A affinity [15]. Similarly, the binding of NRP-2 to VEGFR-3 increases the VEGF-C/VEGFR-3 signaling pathway [14]. The co-receptor HSPG is also important to modulate VEGFR signaling. Indeed, HSPG facilitates the interaction of VEGF with VEGFR-2, thus the heparinase, by cleaving HSPG, decreases VEGFR-2 and ERK1/2 phosphorylation induced by VEGF [17]. 

**Figure 1:**
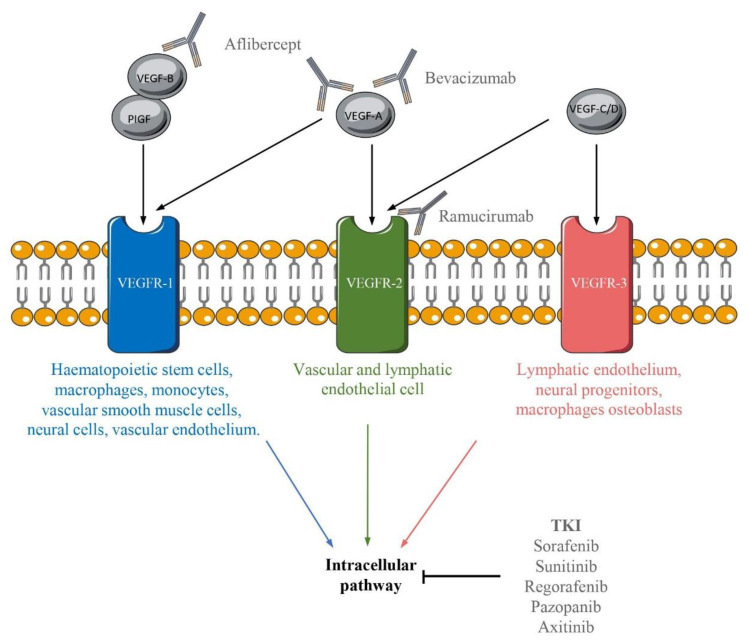
Representation of the different bindings between VEGF/VEGFR and the molecules inhibiting their signaling pathways. The mammalian VEGF family is composed of five members: PIGF, VEGF-A, VEGF-B, VEGF-C and VEGF-D. These different members bind VEGFR: VEGFR-1, VEGFR-2, and VEGFR-3. These receptors are present on the surface of various cells indicated under the receptor. Various inhibitors can target the VEGF/VEGFR signaling pathways.

**Figure 2:**
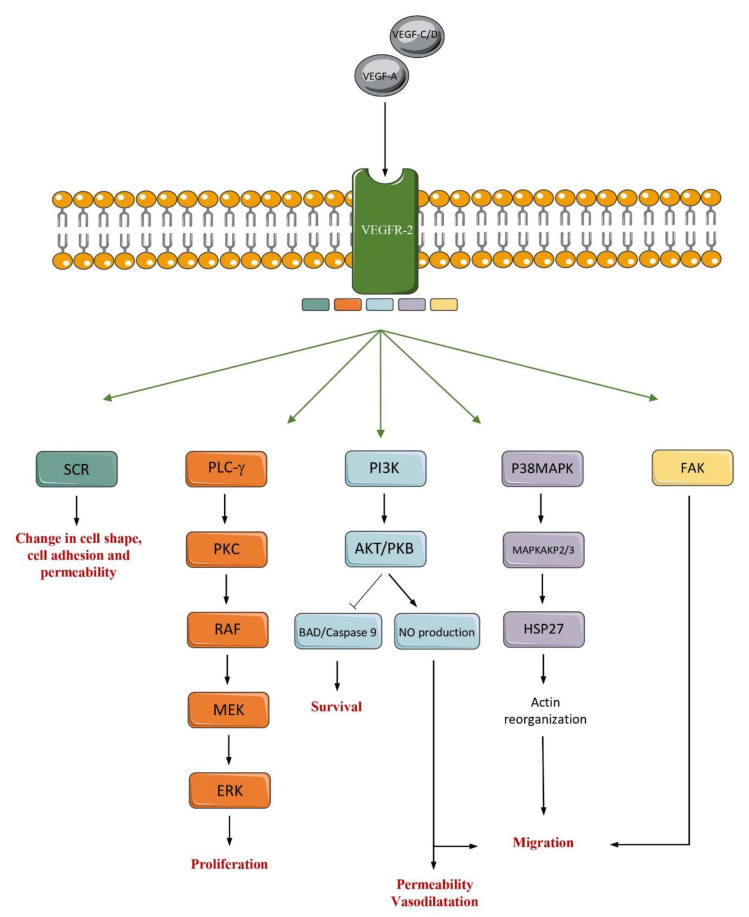
The VEGFR2 signaling pathways in endothelial cells. The VEGFR2 signaling pathways are induced by the binding of VEGF-C, VEGF-E and VEGF-A to VEGFR2. The binding of ligand to VEGFR2 can result in the activation of different pathways such as SCR, PLC-y, PI3K, P38MAPK and FAK. These different pathways control cell shape, cell adhesion and permeability, proliferation, survival, permeability, vasodilatation, and migration.

The receptors of VEGFs are expressed by various cell types (Figure 1) [13,18]. The receptor VEGFR-2 is the most important effector in angiogenesis. This receptor induces many signaling pathways, such as (i) phospholipase-Cγ (PLC-γ)/Protein kinase C (PKC); (ii) p38-Mitogen-activated protein kinase (MAPK); (iii) Phosphoinositide-3-kinase (PI3K)/Protein kinase B (PKB); (iv) SRC and (v) FAK. These signaling pathways induce diverse processes such as migration, permeability, vasodilatation, survival, proliferation, cell adhesion and vascular shape [12] (Figure 2). 

## 3. Insight into the VEGF Signaling Pathways Roles in Angiogenesis

### 3.1. The Angiogenic Switch

When the tumor reaches approximately 2 mm, a process called the angiogenic switch induces angiogenesis. This angiogenic switch activates the formation of new blood vessels in the tumor in order to ensure survival and growth by carrying oxygen and nutrients into the tumor. The angiogenic switch is the result of a shift in the balance between pro- and anti-angiogenic factors in favor of pro-angiogenic factors, mainly induced by hypoxia [5]. Hypoxia induces the activation of numerous genes, including Hypoxia-inducible factor 1 (HIF-1) that is composed of two subunits, α and β. Deficiency in HIF-1α or HIF-1β results in aberrant placental architecture composed of few blood vessels and an early embryogenic lethality in mice [19]. In turn, HIF-1 induces the activation of several genes involved in oxygen consumption, erythrocyte production, angiogenesis, and mitochondrial metabolism [20]. The protein HIF-1 contributes to angiogenesis by inducing the transcription of angiogenic genes such as VEGF, Platelet-derived growth factor (PDGF) and Fibroblast growth factor (FGF) [5]. 

### 3.2. Tumor Blood Vessels Abnormalities 

Tumor blood vessels are relatively distinct from those of normal tissue. Under physiological conditions, newly formed blood vessels rapidly mature and become stable. In tumors, there is a constant growth of tumor blood vessels, creating vessels abnormal in their structures and functions [5].

Normal vasculature is hierarchically structured in arterioles, capillaries, and venules, unlike tumor blood vessels [21]. The basement membrane that usually surrounds endothelial cells, is abnormal in tumors, showing an irregular thickness and potentially holes [21]. In physiological conditions, endothelial cells are joined by adherent and tight junctions, however, in a cancer microenvironment, adjacent endothelial cells are loosely attached to each other’s [22]. Pericytes adhere to endothelial cells, above cell junctions, to mechanically stabilize vessels [22]. In tumor, pericytes are abnormally shaped and loosely attached to endothelial cell. Moreover, an excess of VEGF-A disrupts pericytes recruitment [23]. 

Tumor blood vessels are characterized by an advanced vascular permeability, a high interstitial pressure, and an abnormal blood flow (slow movement and oscillation) [5]. They are also irregularly shaped, dilated, tortuous, leaky, hemorrhagic and have dead ends [5,22]. Tumor blood vessels are also hemorrhagic because of a defective blood vessel barrier. All these characteristics could negatively affect immune response, disrupt immune cells and drug diffusion into the tumor. Angiogenesis inhibition is able to reduce the tumor vascular network, to suppress the formation of new blood vessels [22] and consequently, improves immune cells and drug diffusion to tumors. 

## 4. Cancer Immune Response and VEGF

**Figure 3:**
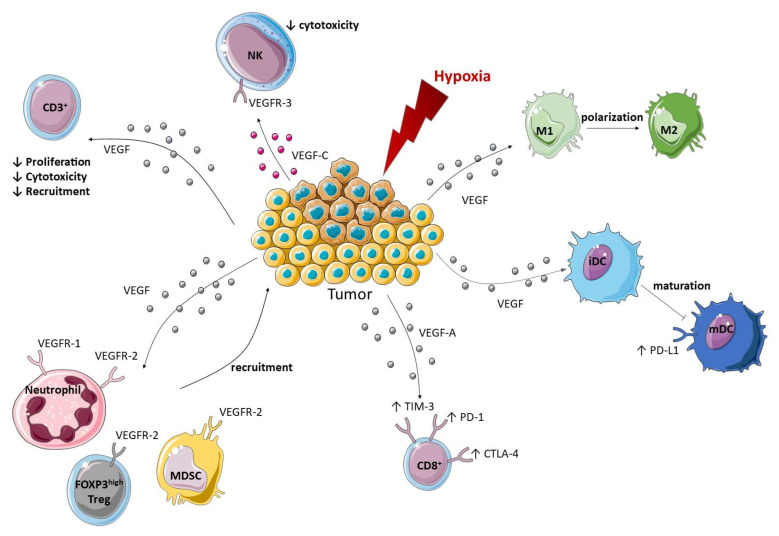
Modulation of the innate and adaptive immune response toward a pro-tumor immune environment by VEGF. An immunosuppressive tumor environment is set up by VEGF thanks to various mechanisms. The first one being the recruitment of neutrophils, Treg and MDSC to the tumor through their expression of VEGFR-1 or VEGFR-2. Polarization of macrophages into an M2 phenotype and inhibition of the maturation of DC by VEGF can also take place in the tumor. Moreover, VEGF up-regulates the expression of checkpoint inhibitors on DC and CD8^+^ T cells while VEGF-C/VEGFR-3 signaling pathway reduces NK cytotoxicity. Finally, VEGF can also reduce the proliferation, cytotoxicity, and recruitment of CD3^+^ T cells. Arrows signify an induction.

The protein VEGF modulates the innate and adaptive immune response directly or indirectly by three different ways: through its interaction with immune cells, by modulating protein expression in endothelial cells, or by modulating vascular permeability (Figure 3). In turn, immunosuppressive immune cells can produce proangiogenic factors and promote angiogenesis, creating a positive feedback loop.

### 4.1. Pro-Angiogenic VEGF Modulates Protein Expression on Endothelial Cells

Immune-cells infiltration within the tumor requires the expression of adhesion molecules at the endothelial cell surface such as intracellular adhesion molecule 1 (ICAM1), vascular cell adhesion protein 1 (VCAM1) and CD34 [24]. Pro-angiogenic molecules can affect the expression of these molecules in endothelial cells, leading to an inhibition of leukocyte adhesion. T cell recruitment is reduced by VEGF that negatively downregulates CD34, ICAM1, and VCAM1 expression on the endothelial cell surface [25,26]. Another report proposes that VEGF does not influence ICAM1 and VCAM1 expression, but rather inhibits their capacity to cluster at the endothelial cells surface [27], thereby limiting their functions. 

In human and mouse tumor blood vessels, VEGF-A, IL-10, and prostaglandin E2 (PGE_2_) increase FasL expression on endothelial cells. Endothelial cells expressing FasL acquire the ability to kill effector CD8^+^ T cell but not FoxP3^+^ regulatory T cells. In conclusion, VEGF participates to CD8^+^ T cell exclusion without affecting Treg, promoting immunosuppression [28]. 

The two chemokines CXCL10 and CXCL11 act as chemoattractant on C-X-C motif chemokine receptor 3 (CXCR3^+^) CD8 and CD4^+^ T cells. To suppress T cell infiltration, VEGF can block CXC-chemokine ligand 10 (CXCL10) and CXCL11 secretion induced by nuclear factor-kappa B (NF-κB) in response to tumor necrosis factor-α (TNF-α) [29]. 

### 4.2. Pro-Angiogenic VEGF Modulates the Innate Immune Response

Depending on the tumor microenvironment (TME), innate immune cells can exert anti or pro-tumor responses. Numerous innate immune cells: Myeloid-derived suppressor cell (MDSCs) [30], macrophage [31], N2 neutrophils [32], Natural killer (NK) [33,34], mast cells (MC) [35,36], promote angiogenesis by producing VEGF. Macrophages [31], N2 neutrophils [32], MDSC [37], and MC [36] can also promote angiogenesis by producing matrix metallopeptidase 9 (MMP-9), a protein known to cleave the extracellular matrix and release heparin bound growth factor such as VEGF-A [38]. Interestingly, deletion of MMP9 in Gr1^+^CD11b^+^ cells reduced their ability to promote tumor growth [39]. These MDSC also produce FGF-2, Bv8 [37], and MC produce FGF-2, IL-8, Transforming growth factor β (TGFβ), TNF-α [35], molecules that can also induce angiogenesis.

Some innate immune cells express VEGFRs. Macrophages [31], neutrophils [40], and dendritic cells (DC) can express VEGFR-1 and VEGFR-2 on their cell surface [41]. Human NK express VEGFR-3 [42]. Human MC also express VEGFR-1, VEGFR-2, NRP-1 and NRP-2 [36] and can be recruited by VEGF [35]. In a mouse OC model, MDSC within the tumor express both VEGFR-1 and VEGFR-2 whereas splenic MDSC express only VEGFR-1 [43]. 

#### 4.2.1. Macrophages

In many cancers, such as BC, thyroid cancer and Hodgkin’s lymphoma, the presence of Tumor-associated macrophage (TAM) is associated with a poor prognosis [44,45,46]. In vitro, VEGF increases their polarization toward an M2 phenotype and their ability to migrate [31]. 

#### 4.2.2. Natural Killer

Patients with surgically resected NSCLC possess NK that produce VEGF and PlGF [33]. Moreover, patients with squamous cell carcinoma (SCC) have a higher VEGF and PlGF secretion comparing to controls [33]. The stimulation of NK with TGFβ-1 increases VEGF and PlGF production [33]. Peripheral NK subject to hypoxia also produce VEGF-A [34]. 

Recently, several studies have been performed to decipher the role of NK in tumor angiogenesis. It was shown that STAT5-deficient NK are able to produce increased levels of VEGF-A, suggesting that STAT5 is a repressor of NK-induced angiogenesis [47]. Secondly, the inhibition of HIF1-α in NK cells slows tumor growth [48]. Interestingly, it has been demonstrated that the binding of VEGF-C on VEGFR-3 present on the NK cell membrane induces a signalization that reduces NK cytotoxicity [42]. In conclusion, NK cells are able to trigger angiogenesis in tumor under specific circumstances but also see their cytotoxic functions reduced by VEGF signaling. 

#### 4.2.3. Neutrophils

Neutrophils play a key role in angiogenesis and in the angiogenic switch initiation. Indeed, the depletion of neutrophils inhibits the initiation of the angiogenic switch in a mouse model of pancreatic multistage carcinogenesis. Murine neutrophils express VEGFR-1 and low levels of VEGFR-2, whereas human neutrophils do not express VEGFR-2 [40]. At steady state, VEGF-A induces CD46d^+^ VEGFR1^high^ CXCR4^high^ neutrophils recruitment to tissue, through VEGFR-1 and VEGFR-2 signaling pathway [40]. Human neutrophils stimulated by VEGF-A show an increased phosphorylation of ERK, suggesting that VEGF-A activates VEGFR-1 signaling in neutrophils [40]. Interestingly, the anti-VEGFR2 antibody (DC101) therapy increases the expression of CX3CL1 that induce Ly6C^lo^ monocytes infiltration, in turn these cells produce CXCL5 that induces immunosuppressive Ly6G+ neutrophils recruitment as a resistance mechanism [49]. 

#### 4.2.4. Mast Cells

In some cancer such as thyroid, gastric, and Hodgkin’s cancers, MC are pro-tumorigenic whereas they are anti-tumorigenic in BC [36]. They accumulate within the tumor before the onset of angiogenesis and reside near blood vessels [50]. Mice deficient for MC have reduced angiogenic and metastatic capacities [51,52]. The constant production of VEGF-A by MC [53] can be boosted by FcεRI, IL-6 [54], IL-9 [55], PGE2 [56], Corticotropin releasing hormone [57] and adenosine [53,58]. The IL-6-induced VEGF production is mediated through a STAT3 dependent signaling pathway [54]. In RCC, MCs support angiogenesis through a PI3K/AKT/GSK3β/Adrenomedullin signaling pathway that drives VEGF expression [59]. 

#### 4.2.5. Myeloid Derived Suppressor Cells

These myeloid cells are a known pro-tumor innate immune population promoted by the tumor. Some studies have shown that MDSC infiltration within the tumor is correlated with an increase of the intratumor VEGF concentration [37]. In a glioblastoma and in a pancreatic-ductal adenocarcinoma mice models, the accumulation of MDSC is associated with the presence of VEGF [60,61]. Within the tumor, hypoxia induces the release of VEGF by MDSCs directly or indirectly through TGFβ or adenosine production. Interestingly, VEGF is able to promote MDSC infiltration within the tumor, creating a positive feedback loop [37]. Within the tumor, MDSCs express VEGFR-2, unlike MDSCs from the spleen, suggesting that MDSC infiltration into the TME depends on the VEGFR-2 signaling pathway. Accordingly, an anti-VEGFR-2 blocking antibody decreases the migratory capacity of MDSCs [43]. A deficiency in VEGF-A results in a decrease of MDSCs and an increase of CD8^+^ T cells within the tumor. Interestingly, the use of anti-Gr1 antibody also induces an increase of CD8^+^ and CD4+ T cells within the tumor, suggesting that VEGF-A reduces the infiltration of cytotoxic T lymphocyte through MDSC [43].

Tumor resistance to anti-VEGF is dependent on MDSC in some models. The thymoma cancer model EL4 is resistant to anti-VEGF (mAb G6.23) and displays an enrichment in MDSC in the tumor. In this model, combination of anti-VEGF and anti-Gr1 reduces tumor growth while monotherapies are weakly efficient [62]. 

#### 4.2.6. Dendritic Cells

Dendritic Cells express VEGFR-1 and VEGFR-2 and VEGF has the ability to inhibit the migration of mature DC through a VEGF-R/RhoA-cofilin1 pathway [41]. This protein also has the ability to upregulate the expression of the checkpoint inhibitor PD-L1 on the DC’s surface [24,63]. 

In patients with breast, head, and neck, or lung cancer, an increased number of immature DC has been associated with high plasma VEGF concentration and a more advanced disease [64]. This finding suggests that VEGF could impair the maturation and differentiation of DC. Accordingly, VEGF could alter the in vitro differentiation of human monocytes to DC [65]. The transcription factor NF-κB is important to induce DCs maturation. Signaling through VEGFR-1 is able to block DCs maturation via an inhibition of NF-κB activation [66,67]. 

Contradictory functions have been reported concerning the role of HIF-1α in DCs. On the one hand, DCs lacking HIF-1α are not able to stimulate T cell proliferation, indicating that HIF-1α is essential to induce an adaptive immune response [68]. One the other hand, a constitutive HIF-1α expression in DCs also impaired the induction of a cytotoxic CD8^+^ T cells response, suggesting that HIF-1α limits the induction of an adaptive immune response [69]. When DCs lack HIF-1α, they express lower levels of the co-stimulatory molecules, CD80, CD86 and CMHII, suggesting that HIF-1α plays a pivotal role in the antigen presentation. In contrast, the lack of HIF-1α does not alter the release of cytokines from DCs [68]. However, a constitutive expression of HIF-1α in DCs increases their production of IL-10 and VEGF, amongst others [69]. Such data underline the ambiguous role of HIF-1α and hypoxia on DC.

### 4.3. Pro-Angiogenic VEGF Modulates the Adaptive Immune Response

In T cell development, VEGF plays a negative role. It induces a diminution of CD4^+^ and CD8^+^ T cells by reducing early hematopoietic progenitor cells [70] and can inhibit directly CD3^+^ T cells proliferation as well as their cytotoxicity [71]. Using an anti-VEGFR-2 monoclonal antibody, T cells proliferation is restored, indicating that VEGF inhibits T cells proliferation through a VEGF/VEGFR-2 signaling pathway [71]. Interestingly, T cells are able to secrete VEGF [71].

In the TME, tumor cells produce VEGF-A, which is able to increase programmed cell death 1 (PD-1), cytotoxic T-lymphocyte associated protein 4 (CTLA-4), and T cell immunoglobulin and mucin-domain containing-3 (TIM-3) on the CD8^+^ T cell surface, inducing T cell exhaustion [72], participating in the establishment of an immunosuppressive microenvironment. This phenomenon can be reversed using anti-VEGF-A antibody or sunitinib, a tyrosine kinase inhibitor (TKI) targeting VEGFR [72]. 

The pro-angiogenic factor VEGF interacts with Treg. Indeed, FOXP3^high^ Treg express VEGFR-2 on their cell surface and VEGF increases Treg recruitment at the tumor site, contributing to an immunosuppressive microenvironment [73]. In hepatocellular carcinoma (HCC), Treg’s infiltration is mediated by NRP-1, a co-receptor of VEGFRs [74]. 

## 5. A Whole Range of Anti-VEGF/VEGFR Therapies 

The use of anti-angiogenic molecules is approved in various treatment regimens against cancer. There are different antibodies and tyrosine kinase inhibitors targeting angiogenesis. These molecules target the signaling pathways of different pro-angiogenic factors, inhibiting either angiogenic molecules, receptors, or tyrosine kinases downstream of the receptors. 

### 5.1. Antibodies Targeting VEGF Pathways

Three recombinant proteins targeting VEGF pathways are approved for cancer use: Bevacizumab, Aflibercept, and Ramucirumab. 

Bevacizumab is a humanized IgG1 antibody directed towards all VEGF-A isoforms [75]. This antibody is thoroughly described and has been administered in approximately 3,500,000 patients suffering from cancer [3]. However, two years after its approval by the US Food and Drug Administration for metastatic BC, Bevacizumab was retracted due to an insufficient benefit/risk balance. It has been shown that Bevacizumab leads to a reduction of Treg in a CRC mouse model and in patients suffering from CRC [24,76,77,78] and increases the number of DCs in the peripheral blood [24,77,79]. Accordingly, Bevacizumab also reestablishes the ability of monocyte to differentiate into DC and restores the expression of CD86 and HLA-DR on DCs [65]. In lung, breast, colorectal and glioblastoma tumor mouse models, DC101, a murine anti-VEGFR-2, decreases the interstitial fluid pressure, the vessel diameter and the microvessel density leading to vessel normalization [80]. Similar observations were made in the peripheral blood of patients suffering from kidney cell cancer treated by bevacizumab [76].

Aflibercept is a recombinant protein constituted of two recognition domains: one of VEGFR-1 and one of VEGFR-2 [81]. These two domains are merged with the Fc fragment of an immunoglobulin [81]. This molecule has the ability to bind to VEGF-A, VEGF-B and PlGF [81]. Bevacizumab [3,76] and Aflibercept [81] inhibit the formation of new blood vessels while inducing the normalization of the already formed ones.

Ramucirumab is a human monoclonal antibody directed towards VEGFR-2. The European Medical Agency (EMA) has approved its use in gastric cancer, metastatic CRC, NSCLC, and HCC [82]. Ramucirumab increases CD8^+^ T cell infiltration in the TME [83]. Ramucirumab [83] and Bevacizumab [24,76,77,78] reduce the frequency of Treg in the TME. 

### 5.2. Tyrosine Kinase Inhibitor

There are many TKI such as Sorafenib, Sunitinib, Regorafenib, Pazopanib, etc., approved for cancer treatment. Others, such as Motesanib (NCT00121628), and Sulfatinib (NCT02549937) are still under investigation. Those antibodies induce an inhibition downstream the ligand/receptor recognition (Figure 1) and have the ability to inhibit the signalization of different receptors. Sorafenib and Sunitinib are the two most described. 

EMA approved Sorafenib for the HCC, advanced RCC and differentiated thyroid carcinoma. This TKI targets different receptors such as VEGFR-1/2/3, PDGFR-β, and c-Kit receptor [84]. Sorafenib is also able to disrupt the tumor vasculature, and to reduce microvessel density [85]. Moreover, Sorafenib reestablishes the ability of monocyte to differentiate into DC and restores the expression of CD86 and HLA-DR on DCs [65]. In patients with HCC, Sorafenib is responsible for a reduction of Treg in the peripheral blood [77].

The EMA approved Sunitinib for gastrointestinal stromal tumor, metastatic RCC and pancreatic neuroendocrine tumors. This inhibitor targets VEGFR-1/2/3, PDGFR-αβ, c-Kit receptor, Fms-like tyrosine kinase-3 receptor (FLT3) and receptor encoded by the Ret proto-oncogene [84]. Interestingly, in sunitinib-treated mice, there is a higher infiltration of CD4^+^ and CD8^+^ within the tumor with a lower CTLA-4 and PD-1 expression, preventing T cells exhaustion [86]. In a B16 melanoma mice model, sunitinib increases T cell infiltration by up-regulating CXCL10 and CXCL11 [29]. Ramucirumab [83], Sorafenib [77,87] and Sunitinib [88] reduce the frequency of Treg in the TME. Furthermore, in patients with metastatic RCC, Sunitinib reduces the number of Foxp3^+^ regulatory T cells in the blood [88].

## 6. The Limit of Anti-Angiogenic Therapies

As with most therapies, inhibiting angiogenesis has limits. Angiogenesis is not only involved in cancer but also occurs in physiological conditions. Thereby, normal endothelial cells express VEGFR and inhibiting angiogenesis interferes with numerous physiological processes such as wound healing. For some patients, angiogenesis inhibition induces side effects such as hypertension, fatigue, asthenia, diarrhea, abdominal pain, and serious side effects such as gastrointestinal perforations and hemorrhage [3]. Unfortunately, anti-angiogenic therapies also increase the risk of cerebrovascular arrest and heart attack. 

Targeting angiogenesis is a promising therapy for solid cancers. However, in certain cancer such as advanced-stage RCC and HCC, it has limited benefits and sometimes displays no efficacy as in prostate cancer, BC, or melanoma [89]. For some patients, angiogenesis inhibition lacks efficiency due to intrinsic or acquired resistance. 

The first resistance mechanism described is the redundancy in the angiogenic signaling pathways. Indeed, when one pro-angiogenic factor is inhibited, another may take over [90]. Tumors can also adopt various neovascularization techniques such as co-option [90]. This neo-vascularization modality corresponds to the ability of cancer cells to incorporate and to grow along pre-existing blood vessels. Inhibiting angiogenesis, especially VEGFR-2, may also increase tumor invasiveness and metastasis [91]. To improve anti-angiogenic therapy interest while limiting resistance mechanisms and side effects, combined therapies are under clinical investigation. 

## 7. Combined Therapies

### 7.1. Anti-VEGF and Other Anti-Angiogenic Mechanisms

Angiopoietin-2 (ANG-2) is another player in tumor angiogenesis. This molecule is an antagonist of ANG-1, a molecule that stabilizes vessels. ANG-2 promotes pericyte detachment from the endothelium [92]. Preclinical and clinical studies in glioblastoma showed that when the tumor becomes resistant to anti-VEGF, there is an increase of the Ang-2 level. Dual inhibition of VEGF (cediranib) and ANG-2 (MEDI3617) normalizes the vasculature and reduces tumor growth in comparison with VEGF inhibition alone in a murine glioblastoma model. This combination alters macrophage polarization and increases the M1/M2 ratio [93,94].

HIF-1 also increases the expression of FGF. During angiogenesis, the FGF signaling pathway mainly involves FGF-2 and FGFR-1 and promotes endothelial cell migration and proliferation [95]. The dual inhibition of VEGF and FGF using Brivanib is active in the first and second line in mouse pancreatic neuroendocrine tumors models developing resistance to VEGF inhibition [96]. 

Endoglin (CD105) is a receptor for TGF-β1 overexpressed on the endothelial cells of blood vessels [97]. This molecule is also expressed on tumor cells, mature innate immune cells such as macrophages and mast cells, and on adaptive immune cells such as T-cells [98]. Endoglin plays a role in angiogenesis, its deficiency leads to a hereditary hemorrhagic telangiectasia type1, defined by vascular malformations [99]. Interestingly, endoglin blocking antibodies downregulated VEGF expression [100] and it has been shown that anti-VEGF therapy upregulates endoglin expression in a preclinical model, indicating that endoglin is implicated in anti-VEGF therapy resistance [97]. A clinical trial (NCT01332721) combining an anti-CD105 (TRC105), and Bevacizumab has shown a good tolerance and clinical activity in adults with advanced cancer. 

Angiogenesis is not the only way for tumors to get nutrients and oxygen; another mechanism is vessel co-option. The combination of angiogenesis and vessel co-option inhibition is a prospective therapy. Indeed, in a preclinical model of advanced CRC liver metastasis, inhibiting vessel co-option and angiogenesis have demonstrated a higher efficiency than each therapy individually [101]. 

### 7.2. Anti VEGF and Chemotherapy

Some anti-VEGF therapies are approved in combination with chemotherapies. 

The EMA has approved Bevacizumab for use in combination with chemotherapies including fluoropyrimidine in the treatment of metastatic CRC and paclitaxel or capecitabine in metastatic BC, in advanced or metastatic kidney cancer, in some advanced NSCLC and in glioblastoma [102]. The EMA has approved its use in metastatic CRC in combination with Folfiri (5-fluorouracil and folinic acid) for patients for whom oxaliplatin is not sufficient [103]. Anti-angiogenic therapy allows a greater chemotherapeutic agent delivery within the tumor. However, for some anti-VEGF therapies, the combination does not improve the progression-free survival (PFS) or overall survival (OS), as in mCRC [104]. 

### 7.3. VEGF and Immune Checkpoint Inhibition

The two major immune checkpoints under clinical investigation are CTLA-4 and PD-1. These checkpoints are expressed on T cells and induce T cell anergy, leading to an inhibition of T cell expansion and function [24]. The inhibition of immune checkpoint is a promising strategy approved in some cancers and its association with inhibitors of the VEGF signaling pathways is under investigation in clinical trials. In patients with metastatic melanoma, after a therapy with anti-PD-1 or anti-CTLA-4, VEGF is decreased in responders and increased in non-responders [105]. Angiogenesis reduces T cell infiltration through different processes. This combination favors T cell infiltration by increasing the number of high endothelial venules [106]. While VEGF-A increases the expression of PD-1 on CD8^+^ T cells, anti-PD-1 therapy limits T cell anergy [72]. 

In mouse tumor models, the use of anti-PD-1 or anti-PD-L1 (clone 6E11) with anti-VEGFR-2 (DC101) induces synergistic effects to inhibit tumor growth [107,108] 

Various ongoing clinical studies focus on the combination of anti-PD-1/PD-L1 and anti-VEGF/VEGFR-2 antibodies in lung cancer (NCT02366143), hepatocellular carcinoma (NCT04102098 and NCT03434379), metastatic colorectal cancer (NCT02997228), renal cell carcinoma (NCT024420821), gastric cancer (NCT02572687), carcinoma of urethral epithelium (NCT02443324), biliary tract cancer (NCT02443324), etc. Most studies are still in early stage but encouraging efficacy has been observed in some cancer types [3,109]. Bevacizumab is still under investigation in clinical trials in order to determine new indications or combination therapies with anti-PD-L1 (NCT02420821), anti-EGFR (NCT02759614), or anti-HER2 (NCT00391092) for example [3].

## 8. Conclusions

Angiogenesis is a key element of cancer development as it is needed for a proper oxygen and nutrients supply to the tumor cells. This process involves different molecules, notably, VEGF, which is the best-known family of molecules stimulating angiogenesis. This family of molecules induces changes in tumor vascularization and favors the establishment of a pro-tumor immune environment. It also promotes tolerogenic immune cell types and dampens cytotoxic immune responses. The immunosuppressive Treg and MDSC are increased by VEGF and it induces macrophage polarization toward an M2 phenotype while decreasing LT-CD8^+^ and LT-CD4^+^. Altogether, these data demonstrate a strong pro-tumor role of angiogenesis and VEGF during tumor development. 

Anti-VEGF molecules are approved for cancer treatment with Bevacizumab, an anti-VEGF-A antibody, being the best known and mostly commonly used. The use of Bevacizumab helps normalize tumor vessels, which favors the tumor infiltration by immune cells as well as the delivery of chemotherapies. Tumors poorly invaded by lymphocytes are more resistant to immunotherapies and to some chemotherapies. Therefore, it makes sense to use anti-VEGF treatments to increase the numbers of lymphocytes within the tumor that are then in the best position to respond to treatments such as antibodies blocking immune checkpoints. 

## Abbreviations

ANG-2	Angiopoietin-2
BC	Breast cancer
CRC	Colorectal cancer
CTLA-4	Cytotoxic T-lymphocyte associated protein 4
CXCL10	CXC-chemokine ligand 10
CXCR3+	C-X-C motif chemokine receptor 3
DC	Dendritic cells
EMA	European Medial Agency
FGF	Fibroblast growth factor
FLT3	Fms-like tyrosine kinase-3 receptor
HCC	Hepatocellular carcinoma
HIF-1	Hypoxia-inducible factor 1
HSPG	Heparan sulfate proteoglycans
ICAM1	Intracellular adhesion molecule 1
MAPK	Mitogen-activated protein kinase
MC	Mast cells
MDSC	Myeloid-derived suppressor cell
MMP-9	Metallopeptidase 9
NF-κB	Nuclear factor-kappa B
NK	Natural killer
NRP	Neuropilin
NSCLC	Non-small cell lung cancer
OC	Ovarian cancer
OS	Overall survival
PD-1	Programmed cell death 1
PDGF	Platelet-derived growth factor
PFS	Progression-free survival
PI3K	Phosphoinositide-3-kinase
PKB	Protein kinase B
PKC	Protein kinase C
PLCγ	Phospholipase-Cγ
PlGF	Placental growth factor
RCC	Renal cell carcinoma
SCC	Squamous cell carcinoma
TAM	Tumor-associated macrophage
TGF-β	Transforming growth factor β
TIM-3	T cell immunoglobulin and mucin-domain containing-3
TKI	Tyrosine kinase inhibitors
TME	Tumor microenvironment
TNFα	Tumor necrosis factor-α
VCAM1	Vascular cell adhesion protein 1
VEGF	Vascular endothelial growth factor
VEGFR	Vascular endothelial growth factor receptors
